# A holistic school-based intervention for improving health-related knowledge, body composition, and fitness in elementary school students: an evaluation of the HealthMPowers program

**DOI:** 10.1186/1479-5868-11-78

**Published:** 2014-06-26

**Authors:** Rachel M Burke, Adria Meyer, Christi Kay, Diane Allensworth, Julie A Gazmararian

**Affiliations:** 1 Department of Epidemiology, Emory University, Rollins School of Public Health, Atlanta, GA, USA; 2 HealthMPowers, Norcross, GA, USA

**Keywords:** School-based intervention, Childhood obesity, Physical activity, Nutrition education, Physical fitness, Children

## Abstract

**Background:**

Over the past 30 years, obesity in the United States has increased twofold in children and threefold in adolescents. In Georgia, nearly 17% of children aged 10 – 17 are obese. In response to the high prevalence of child obesity in Georgia and the potential deleterious consequences that this can have, HealthMPowers was founded in 1999 with the goal of preventing childhood obesity by improving health-enhancing behaviors in elementary schools, utilizing a holistic three-year program. This study measures the effectiveness of the HealthMPowers program in improving the school environment, student knowledge, behavior, cardiovascular fitness levels, and Body Mass Index (BMI).

**Methods:**

The present analysis utilizes data from 40 schools that worked with HealthMPowers over the course of the 2012 – 2013 school year (including schools at each of the three years of the intervention period) and provided information on demographics, student knowledge and behaviors, BMI, performance on the PACER test of aerobic capacity, and school practices and policies (measured via school self-assessment with the HealthMPowers-developed instrument “Continuous Improvement Tracking Tool” or CITT), measured at the beginning and end of each school year. Paired two-sample T tests were used to compare continuous variables (e.g., student knowledge scores, BMI-for-age Z scores), while chi-squared tests were used to assess categorical variables (e.g., trichotomized PACER performance).

**Results:**

Students across all grades and cohorts demonstrated improvements in knowledge and self-reported behaviors, with particularly significant improvements for third-graders in schools in the second year of the HealthMPowers program (p < 0.0001). Similarly, decreases were observed in BMI-for-Age Z scores for this cohort (and others) across grades and gender, with the most significant decreases for students overweight or obese at baseline (p < 0.0005). Students also showed significant increases in performance on the PACER test across grades and cohorts (p < 0.0001). Lastly, schools tended to improve their practices over time, as measured via the CITT instrument.

**Conclusions:**

The present report demonstrates the effectiveness of the HealthMPowers program in producing positive change in school policies and practices, student knowledge and behaviors, and student fitness and BMI, supporting the use of holistic interventions to address childhood obesity.

## Background

Childhood obesity is of increasing concern in the United States: over the past 30 years alone, obesity has increased twofold in children and threefold in adolescents [1]. In 2012, nearly one third of children and adolescents were overweight or obese, with almost 17% classified as obese [2]. Though prevalence of overweight and obesity in United States pediatric populations has not significantly increased over the last decade, it also hasn’t significantly decreased [2]. This is highly concerning, given that obesity in childhood is associated with the development in adults of chronic diseases such as diabetes, hypertension, and dyslipidemia; pulmonary and sleep disorders; and emotional or psychosocial problems (rev. in [3]).

Within the United States, Georgia ranked 17th in 2010 [4] in terms of the prevalence of childhood obesity (a decrease from being ranked 2nd in 2007 [5]), with an estimated 16.5% of children 10–17 years old being categorized as obese [4]. In an analysis of 2007 data, compared to children from Oregon (the state with the lowest prevalence of childhood obesity), children from Georgia had over two times the odds of being obese, after adjusting for age, race, SES, and other confounders [5]. Georgia also has a high prevalence of adult obesity, with nearly a third of adults in 2012 being categorized as obese [4]. As adult obesity is often preceded by childhood obesity, this number is unlikely to go down unless childhood nutritional status is improved in Georgia.

Research has shown that activity patterns developed during childhood can be maintained into adulthood, making it even more important to encourage children to adopt health-enhancing behaviors during this critical period [6]. Adopting lifestyle habits like regular physical activity can lower the risk of becoming overweight or obese and developing chronic diseases such as obesity (rev. in [7,8]). Research also suggests that, in addition to offering critical health benefits, fitness and physical activity are related to improved academic performance [9,10].

Though interventions to address the childhood obesity epidemic have been developed for diverse settings, school, particularly elementary school, has often been a popular choice, given that most children spend a substantial portion of their waking hours at school. Schools also provide the opportunity to reach children regardless of their ethnicity or socio-economic status (SES), both of which have been shown to be associated with increased risk of obesity [3,5]. For this reason, much of the research on reducing childhood obesity has involved integrating prevention and/or intervention activities into school curricula. A recent meta-analysis of school-based obesity reduction programs found that most programs target increased physical activity (23%), education regarding nutrition and physical activity (12%), or both (65%) [11]. Some also included environmental modifications (e.g., removal of vending machines selling unhealthy items; 14%) or behavioral education elements (e.g., teaching decision-making or self-esteem; 28%) [11]. These strategies are consistent with evidence-based recommendations to increase children’s physical activity in the school setting and promote healthful attitudes and behaviors with respect to nutrition and activity [12]. The results of this meta-analysis suggested that school-based programs can cause a moderate, but significant, decrease in child body mass index (BMI) [11]. Furthermore, a recent Cochrane analysis suggested that school-based programs can result in increased moderate-to-vigorous physical activity and improved fitness measures in children [13].

To address the problem of obesity among elementary school-age children in Georgia, HealthMPowers was founded in 1999, in collaboration with the Centers for Disease Control and Prevention (CDC), Children’s Healthcare of Atlanta (CHOA), and the Rollins School of Public Health, Emory University. Based on the advice of this council, as well as the results of a needs assessment conducted with teachers and administrators, the decision was made to focus on elementary school students, given the importance of fostering healthy habits early in life, and the potential for students to continue these habits as they grew. The HealthMPowers initiative was started by two parents who wanted to ensure students made healthier choices. This program utilizes evidence-based programming in a unique, integrated, and holistic approach designed to improve the health of an entire elementary school—including students, staff, and administrators, as well as family members. By targeting all of these stakeholders, HealthMPowers reinforces the message of healthy change in accordance with the tenets of Social Learning Theory [14], Ecological Perspectives on Health Promotion [15], and the Theory of Behavioral Intention [16]. School staff can become models for change in students. When families are also engaged by the classroom teacher to address physical activity and nutrition, then all of the most important individuals in a student’s life, including other students, become role models and create a support system for making healthy choices. Further, this program uses a variety of strategies—instruction, screening, social support, policy, and environmental changes—to reach all of these target populations. School staff are trained to assess their own progress and to make personalized action plans, while being supported with resources, technical assistance, and guidance from experts at HealthMPowers.

The HealthMPowers initiative was never designed to be a research study. It was a practice-based initiative that was designed to use data to assist teachers and families in understanding the need for healthier choices among students, and in turn promoting these choices. HealthMPowers has been collecting and sharing the student, staff, and school policy and programming data with participating schools since 2003. Because the data was collected not as part of a research study but to support a process of continuous improvement within each school, results have not been shared with the broader scientific community. However, these results are enlightening given the unique characteristics of this program. Even though no control group has been used, the collective data from all schools over a period of 11 years has demonstrated repeatedly that major improvements in physical activity choices and fitness levels can be made in the students who participate in the initiative. Further, HealthMPowers works with the school staff to improve their understanding of health issues, which enables them to become the primary trainers and advocates for the changes made in school policy and programming, in turn empowering schools to maintain the continuous improvement process even after the three-year engagement with HealthMPowers has ended. While many school-based programs have focused largely on white populations [7], HealthMPowers’s students come primarily from minority backgrounds (53% black, 17% Latino, 24% white) and low SES families, with the average free and reduced lunch rate being 78% (average for 2012 - 2013 over all three cohorts), with the average free and reduced lunch rate being 78%.

The goal of this article is to describe the HealthMPowers program and the impact that it has had on elementary students in schools throughout Georgia, comparing school and student health indicators collected at baseline and measured throughout the duration of the three-year intervention. Specific aims are to: 1) examine the change in student knowledge and behaviors related to healthy eating and physical activity; 2) assess changes in student fitness levels, as measured via performance on the Progressive Aerobic Capacity Endurance Run (PACER) interval running test [17]; 3) determine changes in student health and body composition as measured via anthropometrics (specifically looking at BMI-for-age Z scores); and 4) examine changes in the school health environment, as measured via a tracking tool designed by HealthMPowers but based upon CDC guidelines [18,19]. Taken together, these indicators will provide the reader with an overall picture of the work that can be accomplished through a comprehensive program such as HealthMPowers, particularly with respect to the initiative’s primary goal of improving students’ adoption of healthy behaviors.

## Methods

### The HealthMPowers program (intervention)

The HealthMPowers program is a three-year, school-wide intervention based on evidence-based guidelines established by the CDC to promote healthy eating and physical activity in schools. The key objectives of the intervention are to: 1) increase health, physical activity, and nutrition education as well as physical activity opportunities for students and staff in school; 2) improve student and staff knowledge about healthy eating and physical activity; 3) improve student and staff health behaviors; and 4) improve school health programs, policies, and environments.

A continuous improvement model is used to make these changes, wherein HealthMPowers provides a team from each school with regular trainings (three times per year), access to a HealthMPowers educator for technical assistance, educational resources, and in-school services (including staff health screenings), along with program assessments to implement and sustain improvements over time (Table 1). Consistent messaging on nutrition and physical activity is integrated daily into curriculum and activities by trained school staff, including classroom teachers, special area teachers, nutrition managers, counselors, and administrators. These messages are supported by educational resources such as classroom exercise DVDs, activity booklets, and lesson plans, which HealthMPowers provides to participating schools. Programs such as “Catch a Teacher Being Healthy” and “Catch a Parent Being Healthy” help to reinforce healthy behaviors among these stakeholders and support them to serve as role models for the children. Yearly school-wide events (e.g., themed assemblies and exhibits) are also used to reinforce the messages of healthy eating and physical activity. A School Health Team (comprising, at a minimum, those school staff participating in the team trainings) is designated to champion the process and monitor progress. Data on student fitness, knowledge, and behavior are collected annually by HealthMPowers along with school policy and program data; these are shared with each school to help staff to understand their school environment and motivate them to engage in improving school-wide physical activity and nutrition programs, policies, and environments.

**Table 1:** Description of training, resources, in-school services, and assessment activities provided by HealthMPowers to participating schools

**Program element**	**Description**
*Training*	• School health team of 3–5 individuals
	○ Provide a foundation for implementation and sustainability of the HealthMPowers intervention
	○ Trained school staff are expected to train colleagues
	○ HealthMPowers will provide on-site training of staff for schools who request it, but online recorded trainings are always available to boost team members’ skills
	• Three HealthMPowers trainings each year to provide professional guidance and technical assistance regarding:
	○ Assessing baseline health school programs, policies, and environment
	○ Developing an action plan
	○ Implementing HealthMPowers materials and curriculum
	• Annual refresher training of school staff on collection of height, weight, and fitness data
*Resources*	• Resources and teaching aids consistent with Georgia Performance Standards and Common Core State Standards
	○ Facilitate integration of health education and physical activity instruction into the school day
	○ Includes activity booklets, classroom exercise DVDs, integrated lessons, family-based reinforcement activities
*In-School Services*	• Student-focused: School-wide assemblies, student classroom lessons, 10-station exhibit about the human body (1 per year)
	• Staff-focused: Staff wellness support
*Assessment*	• Data collected and analyzed by HealthMPowers and school staff
	○ First year: baseline data on school practices and policies collected to determine strengths and areas for improvement; student knowledge, self-reported behaviors, and fitness data collected at the beginning and at the end of the school year
	○ Subsequent years: data on school practices and policies collected to determine strengths and areas for improvement; student knowledge, self-reported behaviors, and fitness data collected at the beginning and end of the school year
	• Schools may also request yearly assessments of staff health risk and staff fitness
	• Yearly report from HealthMPowers to each school; also provides assessment tools that schools can use after completing the program

HealthMPowers has an actual cost of $30 per student (as determined by HealthMPowers calculations), but the majority of this cost is subsidized by various grants and sponsors. Schools are asked to provide release time for their School Health Team members to attend three full-day trainings annually as their financial investment into the program. Table 1 lists the training, resources, services, and assessments provided by HealthMPowers to participating schools.

### Study design and school population

Schools self-select into the HealthMPowers program, which they may learn about from the school district, other schools, or directly from HealthMPowers. Though the HealthMPowers program design is based on the Centers for Disease Control and Prevention’s published guidelines [18,19], the specific combination of elements, as well as the pace of their implementation, has not been formally tested for efficacy or effectiveness.

This paper utilizes data collected during the 2012 – 2013 and 2011 – 2012 school years for three recent cohorts of HealthMPowers elementary schools in different stages of program implementation—those initiating partnerships in 2010, 2011, and 2012—as representative of the HealthMPowers experience. Knowledge and behavior indicators are measured at the beginning (September – October) and end (April – May) of each year (as well as at baseline, prior to initiation of the HealthMPowers program). All fourth and fifth grade students were measured on fitness indicators in 2012 – 2013, with fifth graders also measured in 2011 – 2012; over the 2011 – 2013 school years, knowledge data was collected for all third grade students in the 2011 and 2012 cohorts as well as for fourth graders from the 2010 cohort, while over the 2011 – 2012 school year, data was also collected for third though fifth graders in the 2011 and 2010 cohorts. Individual students were not followed for more than one year at a time.

During the 2012 – 2013 school year, 61 schools worked with HealthMPowers using its comprehensive three-year program model; these schools were spread across 19 districts. The intervention reached over 39,272 students and their families, along with over 2,604 school staff. Schools self-select to work with HealthMPowers and may pursue various funding sources in order to subsidize the service fees. Of these schools, only those which collected all data measures listed below (40 schools) were included as a part of this study.

As the program was administered at the school level, all students within each participating school are considered to be exposed, though not all measures were available for all students. All students with available data were included, though missing values were assigned for non-biologically plausible values, as described below under “Student-level Indicators”.

### Data collection and outcome evaluation (measures)

#### Student-level indicators

Student-level progress is assessed in several domains: knowledge, behavior, and fitness. Knowledge and behavior are assessed via a student questionnaire administered at the beginning of the school year (during the pre-intervention period, September – October) and at the end of the school year (April – May). “Correct” or “optimal” responses (based on whether or not students meet the Georgia health education state standards and national health recommendations [20-24]) accrue points, while “incorrect” or “sub-optimal” responses do not, and overall performance is measured via the mean percentage of points accrued out of the total possible points by section and overall. Student progress is also measured by whether or not students improve their scores over the course of the school year. For the 2012 cohort, data was available for third-graders over the 2012 – 2013 school year. For the 2011 cohort, data was available for third-graders over the 2012 – 2013 school year and for third – fifth graders over the 2011 – 2012 school year. For the 2010 cohort, data was available for fourth graders over the 2012 – 2013 school year and for third – fifth graders over the 2011 – 2012 school year.

Fitness is measured both by body mass index (BMI) and by performance on the Progressive Aerobic Cardiovascular Endurance Run (PACER) test measuring cardiovascular fitness. BMI is calculated from student height (inches) and weight (pounds), as measured by HealthMPowers-trained school personnel at the beginning and end of the school year. These values are converted to age- and sex-specific BMI-for-age Z scores, using the CDC growth curve references and calculated with a SAS macro created by CDC [25]. For the purposes of this analysis, “pre” and “post” dates were fixed across schools at September 15 and April 15, respectively, although in practice, testing dates often varied by several weeks from school to school. This was necessary to reflect changes in the students’ ages, as not all schools provided complete data on “pre-test” dates. Student performance on the PACER is measured in absolute number of laps completed, and students are assessed on whether their performance improved, decreased, or remained the same over the course of the school year. For the 2012 cohort, BMI and PACER data was available for fourth- and fifth-graders for the 2012 – 2013 school year. For the 2011 – 2012 cohort, BMI and PACER data was available for fourth- and fifth-graders for the 2012 – 2013 school year. For the 2010 cohort, BMI and PACER data was available for fourth- and fifth-graders for the 2012 – 2013 school year, and for fifth-graders for the 2011 – 2012 school year.

#### School-level indicators

Self-assessment of school-level progress is assessed by a tool created by HealthMPowers to measure different facets of program implementation. Schools use this “Continuous Improvement Tracking Tool” (CITT) to self-assess their policies and programs both at baseline and at the end of each school year (April – May). CITT data is available for the 2012 – 2013 school year for all cohorts, for the 2011 – 2012 school year for the 2011 and 2010 cohort, and for the 2010 – 2011 school year for the 2010 cohort. The CITT instrument, set up as a rubric, is administered during trainings and is completed by the School Health Team. The rubric includes a number of indicators designed to measure the following categories of school health in regard to physical activity and nutrition: student health education (e.g., number of hours per year of health instruction), physical education (PE) and physical activity (PA; e.g., frequency of each), staff wellness (e.g., dedicated time at staff meetings), family involvement (e.g., provision of health information to families), activities of the School Health Team (e.g., assessment activities), and school environment (e.g., implementation of physical or cultural changes such as removing unhealthful food from vending machines). For each indicator in the various categories, there are four degrees of implementation (Needs Improvement, Making Progress, Healthy School, Model Healthy School). The descriptions of these degrees are based on the intensity and comprehensiveness of activities within each indicator. For instance, within the “school environment” category, a “Model Healthy School” would have implemented at least four policy changes since beginning to work with HealthMPowers, e.g., stocking vending machines with healthy items, disallowing the withholding of physical activity as a punishment, disallowing the use of food as a reward or punishment. The School Health Team self-rates each item on the CITT, assigning their school to a level based on the descriptions of each level. For the present report, we consider mean scores on each category, as well as a mean overall “School Health Score”, created by summing the scores for each facet. Given that the CITT is specifically designed as a self-assessment tool, results should be interpreted as subjective measures of school progress in regard to their baseline assessment.

### Data management, cleaning, and statistical analysis

Data were entered into Microsoft Excel spreadsheets. Excel and SAS, v. 9.2 (Cary, NC) were used for data analysis. Data cleaning was performed, and biologically implausible values for BMI were set to “missing”. Observations with biologically implausible values for changes in height or weight were also excluded. Schools that were missing complete data on one or more measures of interest were excluded from the analysis. These totaled 21 of 61 schools, leaving 40 schools for the present analysis.

School self-assessment of practices and policies was measured via the tracking tool described above, with mean yearly scores compared year-on-year; a paired two-sample t test was used to assess changes. Improvement in knowledge, behaviors, and self-efficacy was assessed via questionnaire, with means (stratified on grade, cohort, and data year) compared using paired two-sample t tests. Changes in student BMI and PACER performance were also assessed by comparison of means using paired two-sample t tests and stratifying on gender, grade, cohort, and data year. Differences of improvement category for the PACER test (i.e., improve, maintain, or decrease score) were assessed via Chi-Squared tests. To account for multiple comparisons, an alpha level of 0.0005 was considered appropriate.

### Ethical approval

The HealthMPowers program was not designed as a research study, but rather as a comprehensive practice-based initiative based upon strategies recommended by the Centers for Disease Control and Prevention (CDC) for improving healthful behaviors around physical activity and nutrition in school settings. Because the data for the present analysis was fully de-identified and not originally collected for research purposes, this analysis was declared not human subjects research, and did not require IRB approval.

## Results

Overall, 40 elementary schools with data on all indicators of interest were included in the present analysis: 12 schools that began the HealthMPowers program in the 2012–2013 school year (“2012 cohort”), 22 schools that began the HealthMPowers program in the 2011–2012 school year (“2011 cohort”), and 6 schools that began the HealthMPowers program in the 2010–2011 school year (“2010 cohort”). The mean number of students per school ranged from 533 (2010 cohort) to 609 (2011 cohort) (Table 2). Included schools were not significantly different from non-included schools in terms of total number of students or number of students by race; the mean percentage of students receiving free or reduced lunch was moderately higher among the non-included schools (87.5% vs. 78.2%; p = 0.025), though the mean number of students receiving free or reduced lunch was not significantly different by inclusion status (p = 0.876).

**Table 2:** **2012**–**2013 demographics of 40 schools participating in HealthMPowers program and providing data on child knowledge and behavior, fitness (BMI, PACER test), HealthMPowers resource usage, and school policies and practices**

**School cohort**	**Year 1 (n=12)**	**Year 2 (n=22)**	**Year 3 (n=6)**
**School characteristic**			
Number of students K – 5 *(mean [total])*^1^	563 (6756)	609 (13,394)	533 (3197)
Free/Reduced Lunch *(%)*^2^	69%	79%	92%
# of K – 5 teachers *(mean)*^3^	38	43	36
Race/Ethnicity *(%)*^4^			
White	51%	18%	36%
Black	26%	61%	47%
Hispanic	1%	14%	14%
Asian/Pacific islander	18%	2%	1%
Native American	0%	0%	0%
2 or more races	4%	5%	2%

^1^Mean number of students per school (total number of students per cohort).

^2^Percentage of students per cohort receiving free or reduced lunch.

^3^Mean number of K – 5 teachers per school.

^4^Percentage of students per cohort identifying as each race/ethnicity.

Demographics varied by cohort, with the percentage of children receiving free or reduced lunch ranging from 69% (2012 cohort) to 92% (2010 cohort). The percentage of children in the 2012 cohort who were white (51%) was greater than the corresponding proportion in the other cohorts (18% and 36% for cohorts 2011 and 2010, respectively). The 2012 cohort also had a lower percentage of children reporting black race (26%) as compared to the other cohorts (61% and 47% for the 2011 and 2010 cohorts, respectively).

**Figure 1:**
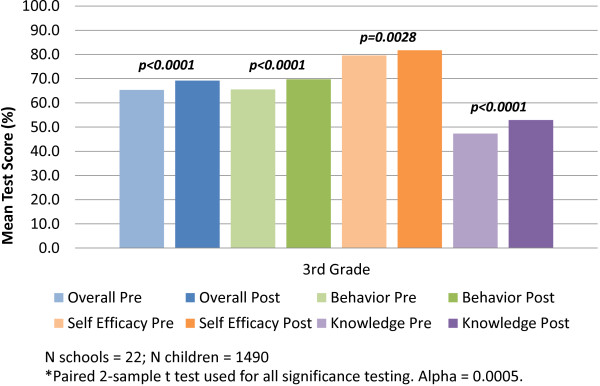
**Changes in self-reported student knowledge and behaviors.** Changes in student performance on a test measuring healthful behaviors, self-efficacy, and nutrition- and physical activity-related knowledge, administered during the fall (pre) and the spring (post) of 2012 and 2013, respectively, for students from a cohort of 22 schools starting the HealthMPowers program in 2011–2012 and providing data on student knowledge and behavior, fitness measurements (BMI, PACER test), HealthMPowers resource usage, and school policies and practices.

Overall teacher-reported usage of HealthMPowers resources (compliance) was high, particularly for student and teaching resources such as school-wide assemblies (91-100%, depending on cohort), model lessons (95-100%), and educational DVDs (91-100%) (Table 3). Student and teacher newsletters were utilized less frequently, though family newsletters were popular among schools.Across all cohorts, significant improvements in student knowledge, behavior, and self-efficacy were seen generally across categories, particularly for schools in their first or second year of the program. Increases in knowledge were generally higher than increases in other categories. For students measured in 2012–2013 during the second year of the HealthMPowers program, mean student scores significantly increased over the course of the school year, with the greatest magnitude of change seen for knowledge (p < 0.0001; Figure 1). Changes were less significant for the 2010 cohort of 4th graders completing their third year of the program (p = 0.3884 for overall change; 2012–2013), as well as 5th graders from the 2010 cohort measured in their second year (p = 0.0025 for overall change; 2011–2012); these students generally had higher scores to begin with, as compared to third graders. (Data available upon request).

**Table 3:** **2012**–**2013 Reported usage of HealthMPowers resources (any usage vs. none), by cohort year in the program for the 40 schools participating in the HealthMPowers program and providing data on student knowledge and behavior, fitness measurements (BMI, PACER test), HealthMPowers resource usage, and school policies and practices**

**School cohort**	**Year 1 (n = 12)**		**Year 2 (n = 22)**		**Year 3 (n = 6)**	
**Resources**	Resource usage (frequency, percentage)*					
*Student and teaching resources*						
School-wide assemblies or body walk	11	(92%)	20	(91%)	6	(100%)
Topical books	11	(92%)	17	(77%)	6	(100%)
Educational exercise DVDs	12	(100%)	20	(91%)	6	(100%)
Activity kits and materials	12	(100%)	21	(95%)	6	(100%)
Model lessons and teaching material	12	(100%)	21	(95%)	6	(100%)
Student newsletters	10	(83%)	17	(77%)	5	(83%)
Student health advocate club^†^	N/A	N/A	N/A	N/A	1	(17%)
*Family resources*						
Family newsletters	12	(100%)	21	(95%)	6	(100%)
Family activities^†^	N/A	N/A	N/A	N/A	4	(67%)
*Staff resources*						
Health/Fitness screenings	12	(100%)	18	(82%)	5	(83%)
Trainings	12	(100%)	20	(91%)	5	(83%)
Posters	11	(92%)	19	(86%)	5	(83%)
Teacher newsletters	10	(83%)	17	(77%)	5	(83%)
Website	11	(92%)	15	(68%)	5	(83%)

*Each year, schools report whether or not they utilized each available HealthMPowers resource. Resources have been grouped into the above categories. Frequencies above refer to number of schools per cohort reporting any usage of a resource in the indicated category.

^†^These activities were offered only to Year 3 schools and select Year 2 schools. Therefore, only usage by Year 3 schools is reported.

**Figure 2:**
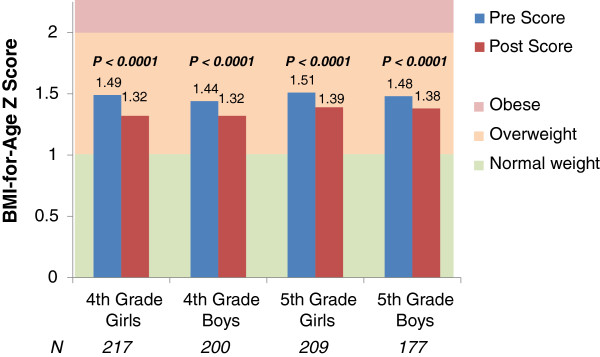
**Changes in BMI-for-age Z score for students overweight at baseline.** BMI-for-age Z score, measured during the fall (pre) and the spring (post) of 2012 and 2013, respectively, for students overweight at baseline (fall measurement), from a cohort of 22 schools starting the HealthMPowers program in 2011–2012 and providing data on student knowledge and behavior, fitness measurements (BMI, PACER test), HealthMPowers resource usage, and school policies and practices. Paired two-sample t test used to evaluate changes in pre vs. post BMI-for-age Z score. Alpha=0.0005.

**Figure 3:**
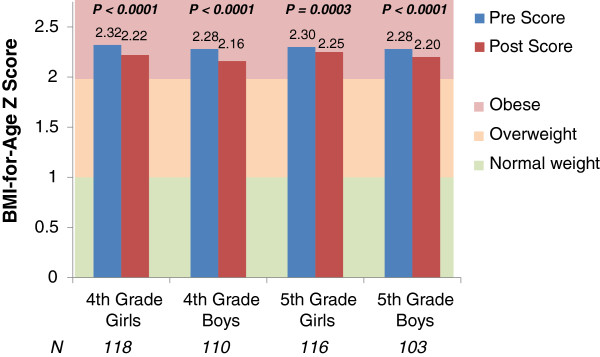
**Changes in BMI-for-age Z score for students obese at baseline.** BMI-for-age Z score, measured during the fall (pre) and the spring (post) of 2012 and 2013, respectively, for students obese at baseline (fall measurement), from a cohort of 22 schools starting the HealthMPowers program in 2011–2012 and providing data on student knowledge and behavior, fitness measurements (BMI, PACER test), HealthMPowers resource usage, and school policies and practices.

Program impact on student body composition and fitness levels was measured via changes in BMI-for-age Z score and performance on the PACER fitness test. Given that it has been shown that gender and baseline weight status may affect changes in fitness, we stratified BMI-for-age results on cohort, grade, gender, and baseline weight status. For students in the 2011–2012 cohort, the magnitude of change during the 2012–2013 school year was greatest for students that were overweight or obese at baseline, with significant decreases in BMI-for-age Z score observed for both boys and girls in 4th and 5th grades (Figures 2–3). This pattern was repeated for other cohorts and years. Generally, the magnitude of BMI-for-age Z change was slightly greater for 4th graders. (Data available upon request). Increases in cardiovascular fitness were measured via performance on the PACER test, and were significant for all grades and cohorts (Table 4). Fifth-graders from the 2010 cohort had a greater percentage of students improve than fourth-graders from the same cohort during the 2012–2013 year.

**Table 4:** Changes in student performance on the PACER test, measured in fall (pre) and spring (post), by grade, year, and cohort, for 40 schools providing data on student knowledge and behavior, fitness measurements (BMI, PACER test), HealthMPowers resource usage, and school policies and practices

	**Average # of laps completed**			**Student-level change (pre to post)**			
	**Pre**	**Post**	**P-value**^**†**^	**Improved**	**Maintained**	**Decreased**	**P-value (4th vs. 5th)**^**†**^
**2012-2013 Data**							
* 2012-2013 Cohort*							
4th grade	19.0	21.4	***<0.0001***	481 (57%)	62 (7%)	303 (36%)	0.02
5th grade	22.5	24.5	***<0.0001***	490 (56%)	98 (11%)	294 (33%)	
* 2011-2012 Cohort*							
4th grade	22.2	24.7	***<0.0001***	876 (62%)	104 (7%)	439 (30.9%)	0.48
5th grade	25.5	28.3	***<0.0001***	839 (62%)	83 (6%)	423 (32%)	
* 2010-2011 Cohort*							
4th grade	17.5	25.3	***<0.0001***	245 (66%)	36 (10%)	89 (24%)	***<0.0001***
5th grade	19.1	26.3	***<0.0001***	259 (81%)	21 (7%)	40 (13%)	
2011-2012 Data*							
* 2010-2011 Cohort*							
5th grade	16.6	19.0	***<0.0001***	188 (65%)	35 (12%)	66 (23%)	

*Data not available for 4th graders in the 2010–2011 cohort in 2011–2012. Data not available for 2011–2012 for the 2011–2012 cohort.

^†^Paired two-sample t-tests used to evaluate significant differences in pre as compared to post measurements. Chi squared tests used to evaluate differences in student-level change by grade. Alpha = 0.0005.

At the school level, we observed improvements in school-wide health practices and policies over time, with scores reaching the target zones set by HealthMPowers (Table 5 shows data for the 2011 cohort; other data available upon request). Scores for Family Engagement and School Health Team Activity showed the greatest increases, by 65% and 71% from baseline, respectively, for this cohort.

**Table 5:** Mean (SD) scores in self-reported scores for school policies and practices, from 2011–2012 to 2012–2013, for the cohort of 22 schools starting the HealthMPowers program in 2011–2012 and providing data on student knowledge and behavior, fitness measurements (BMI, PACER test), HealthMPowers resource usage, and school policies and practices

	**Student health programming**	**Physical education & physical activity**	**Staff wellness**	**Family engagement**	**School health team**	**School environment**	**Overall school**
Baseline*	30.1 (9.3)	27.8 (9.2)	13.2 (4.3)	12.1 (5.3)	10.1 (6.3)	18.2 (7.4)	111.5 (31.8)
**2011-2012 (post)**	34.4 (6.0)	26.6 (4.9)	18.9 (4.9)	16.7 (4.8)	15.3 (6.2)	19.3 (5.7)	131.2 (20.3)
**2012-2013**	38.8 (8.1)	34.2 (7.2)	18.6 (4.5)	19.9 (5.5)	17.2 (6.8)	25.8 (8.3)	154.5 (26.7)
**P-value (baseline to 2012–2013)**^**†**^	0.0021	0.0057	***0.0008***	***<0.0001***	0.0012	0.0025	***<0.0001***

*Baseline measurement is taken in 2011–2012 before the school begins implementing the HealthMPowers resources. Schools self-rate each year during the fall and again during the spring, describing their policies and practices. These are then scored against a target zone. Domains measured include student health programming (e.g., whether students are engaged in regular health education), physical education and activity (e.g., frequency with which PE class is offered), staff (e.g., whether health is discussed at staff meetings), family (e.g., whether families are provided with health information), School Health Team (e.g., whether a team has been convened, has had meetings), and school environment (e.g., has made policy or environmental changes such as removing vending machines selling sugar-sweetened beverages). Scores are totaled to come up with an overall assessment of how well the school’s policies and practices promote health.

^**†**^Paired two-sample t test. Alpha = 0.0005.

## Discussion

In this evaluation of 40 schools participating in the HealthMPowers program, we observed significant improvements over time in student health-related knowledge, self-efficacy, and behaviors; student fitness as measured by BMI-for-age and performance on the PACER test; as well as in school policies and practices. Improvements were stronger in the first two years of the program as compared to the third year. We hypothesize several potential explanations for this difference: there could be a threshold reached beyond which improvement is more difficult to achieve, or the small sample size in this cohort could contribute to random error which could obscure a true effect.

Although our study design, without a control group, precludes direct comparison with some other programs studied, our results are comparable to those found by similarly comprehensive programs that incorporate not only classroom health education lessons and increased opportunities for physical activity, but also involve school staff and families in promoting and reinforcing positive behaviors. One program consistent with this philosophy is the *Healthy Schools Program*, a four-year program that involves the formation of a “school wellness council”, completion of an assessment, action planning, and technical assistance and implementation support (online tools, “train the trainer” sessions for teachers) to enable improvements in school environment to support health (e.g., PA opportunities for students, staff wellness, availability of nutritious food) [26]. An evaluation of that program in 2012 showed that participating schools demonstrated significant improvements in employee wellness, school meals, health education and physical activity, as well as policies and systems [26]; however, no assessment was made of student-level knowledge or fitness outcomes and, as in the present report, this analysis lacked a control group. Another program involving the creation of school-specific action plans was assessed using a quasi-experimental design in 18 Swedish schools; this study indicated significant improvements of school policies and practices, but did not show any significant differences between intervention and control groups in student-level outcomes such as behavior or BMI [27]. The *Alberta Project Promoting active Living and healthy Eating* (*APPLE*) program utilizes “School Health Facilitators”, individuals placed in each school to address the specific school environment—including facilitators and barriers to healthful policies, practices, and behaviors—and engage stakeholders at all levels, from parents, to students, to staff [28]. A 2012 evaluation of this three-year intervention, in comparison to a set of randomly selected non-intervention schools in Alberta, found not only improvements in student behaviors (e.g., increased fruit and vegetable consumption, increased physical activity), but also decreases in weight [28]. The *Action Schools! BC* intervention similarly provides support to schools to customize the inclusion of health education and physical activity promotion into school curricula, while also making environmental changes (e.g., modifications to playground equipment) and engaging family and community members [29]. When evaluated in 2011 using a pre-post design similar to our own, this program was shown to increase aerobic fitness in students over the course of seven months, though few other significant changes were seen in student-level indicators (no significant changes in physical activity or BMI-for-age Z scores, for example) [29]. Other comprehensive school-based programs also showed significant effects across some or all measures, although they were not tailored to specific schools, as were the above examples (and the HealthMPowers initiative). Reviews suggest that, at minimum, effective programs must incorporate increased opportunities for physical activity in addition to physical and health education classes, and that behaviors are easier to change than fitness levels (rev. in [7,13,30]), although changes in student fitness did occur with the HealthMPowers program. (However, it must be noted that PACER results are not directly comparable to other measures of fitness such as the one-mile run). Further, a whole-school approach in line with the WHO-recommended health promoting school framework [31,32] appears to generate better student outcomes than a focused intervention where activities are confined to the classroom or physical education class.

This analysis has at least four strengths. First, student-level data was obtained not only on knowledge and behaviors, but also on BMI and aerobic capacity (as measured by the PACER test), enabling analysis of the program’s effect on student body composition and fitness. Second, student-level data were supplemented by school-level assessments of policies and practices, providing insight into how school environments change over the course of the HealthMPowers program. Furthermore, we were able to include data from multiple cohorts of schools, enabling us to look at the effect of the program over time. Lastly, the fact that this was an effectiveness study rather than an efficacy study strengthens the applicability of the findings to everyday practice. These results represent the effect of the HealthMPowers program as implemented in a real-world setting with a diverse student body, enhancing the generalizability of these results to other public school settings.

Despite these strengths, there are at least five limitations. First, and most significantly, although baseline data was collected for each school, there were no control schools, introducing the possibility that observed differences were due to secular changes over time. However, given that significant changes were observed across cohorts (i.e., year-on-year changes were observed regardless of start year), we feel that this is not likely to explain our findings. Additionally, although changes in student height and weight are to be expected over time, utilization of a BMI-for-age Z score takes into account this natural variation by age. Furthermore, recent data suggests that prevalence of obesity (defined, for children, by BMI-for-age measures) among elementary-school-aged children is remaining steady over time [2], making secular changes an unlikely explanation for the decreases seen in BMI-for-age Z score. We did do a sensitivity analysis with change in BMI, and found a mix of increases and decreases in BMI among the different strata of children overweight or obese at baseline (magnitude range: −0.28 – 0.51, mean change 0.16). All changes were nonsignificant. Given the age of the children (mean age 10.8), we would expect a slight increase in BMI over the course of the school year [33]. While student aerobic capacity will also increase naturally over time, the fitness recommendations based on PACER performance don’t change between the ages of 10 and 11 (mean age in our cohort was 10.8 years), and average student performance (upon which criteria are based) changes very little from ages 11 to 12 [34]. A second limitation is that due to the data collection design, we were unable to follow individual students from year to year. Thirdly, not all participating schools provided data on all indicators of interest; however, schools that did not provide data were similar demographically to schools that did provide data, with the exception of the mean percentage of students receiving free or reduced lunch (p = 0.025; although the mean total number of students receiving free or reduced lunch was not significantly different by inclusion status: p = 0.878). Another limitation is that the measurement of school-level changes was performed with the CITT instrument, which has not been separately validated despite having been used by HealthMPowers for over ten years. However, a sensitivity analysis comparing student outcomes between schools with above-average improvement as measured by CITT and schools with below-average improvement did uncover substantial differences in changes in student weight status, which helps to support the validity of the CITT. Specifically, students from above-average improvement schools experienced a −0.08 point decrease in BMI-for-age Z score, as compared to a −0.04 decrease among students from schools with below-average improvement (p = 0.0016). Similarly, students from above-average schools gained only 0.17 BMI points as compared to 0.28 BMI points in students from below-average schools (p = 0.02). Assessment of inter- and intra-rater reliability of the CITT, as well as test-retest reliability, is currently being undertaken at the time of publication; validity studies are also being considered. Lastly, this study is limited by the fact that student behavior data were self-reported, while PACER and BMI data were collected by teachers as opposed to health professionals or study staff. However, HealthMPowers has trained school staff in how to correctly collect PACER, height, and weight data, and also provides annual refresher training to schools. Further, a comprehensive fitness assessment manual was developed by HealthMPowers for the Georgia Department of Education, and an electronic version is available on the Georgia Department of Education’s website. Unfortunately, budget limitations precluded use of accelerometers or pedometers to objectively measure student physical activity.

Although this study contributes to the body of knowledge on childhood obesity prevention, some gaps remain to be addressed. Future research should focus on which specific elements of school-based programs are most effective in changing student behaviors and fitness levels—for instance, what is the contribution of a School Health Team? How does family engagement factor into student-level outcomes? While some randomized trials (e.g., Williamson et al. [7]) have attempted to assess the effect of primary prevention (environmental modification) compared to primary plus secondary prevention (education), few studies have specifically looked at the comparative effectiveness of program elements in detail. Additionally, more studies are needed that follow children enrolled in long-term (more than six-month) interventions over the course of several years. Lastly, given the potential connections between physical fitness and academic performance [9,10], future research should measure academic performance as an outcome of increased levels of physical activity during the school day. Future research on the HealthMPowers program specifically could include following individual children across years, as well as assessing other outcomes such as academic performance.

Considering the HealthMPowers program along the RE-AIM (Reach, Efficacy, Adoption, Implementation, Maintenance) framework also provides a helpful way to assess its overall public health impact [35]. As discussed above, HealthMPowers reached nearly 40,000 students over the course of the 2012 – 2013 school year, representing substantial reach. Although efficacy has not been demonstrated in a randomized controlled trial, the program’s effectiveness in improving student- and school-level health indicators has been shown in the above analysis. Adoption is by definition high given that this was an evaluation of already-participating schools. Further, given that this was an evaluation of an existing program, the results demonstrated reflect the current level of implementation and that level which would be expected in similar settings with similar resources. Maintenance (extent to which a program is sustained over time) is demonstrated with HealthMPowers by the fact that many schools choose to continue working with HealthMPowers even after the three-year program has finished, and the fact that few drop out. An analysis of sustainability conducted early in HealthMPowers’s history demonstrated that two years after its programming ceased in 12 schools, all of the schools continued to provide daily physical activity, annual fitness testing of students, and worksite health promotion activities for school staff. Eleven of the 12 schools continued to engage students in goal-setting and implementing a plan to improve their health-related fitness, to involve families in supporting their child’s self-improvement plan, and to make environmental and policy changes necessary to align health programming and practice [36].

## Conclusions

The present report demonstrates the effectiveness of the HealthMPowers program in producing positive change in self-assessed school policies and practices, student knowledge and behaviors, and student body composition and fitness. This suggests that comprehensive, tailored, and whole school-based programs incorporating health education, physical education, supplemental physical activity, and staff and family involvement can have a significant impact on child health and fitness. In an era where child obesity is of increasing concern, it is critical to identify effective prevention programs. Future research should focus on specific program elements that are most impactful, as well as employ measures to track individual students as they participate in long-term interventions over time.

## Abbreviations

BMI: Body Mass index; CDC: Centers for Disease Control and Prevention; CITT: Continuous Improvement Tracking Tool; PACER: Progressive Aerobic Capacity Endurance Run; SES: Socioeconomic status.

## Competing interests

RMB and JG declare that they have no competing interests. AM is employed by HealthMPowers, and CK is its President, while DA is an emeritus employee.

## Authors’ contributions

RMB was a primary contributor to the study design and analysis plan, cleaned and analyzed the data, and drafted the manuscript. AM contributed to the study design and analysis plan and helped to draft the manuscript. CK contributed to the study design and helped to draft the manuscript. DA helped to draft the manuscript. JG contributed to the study design and analysis plan, and helped to draft the manuscript. All authors read and approved the final manuscript.

## References

[B1] National Center for Health Statistics Health, United States, 2011: With Special Feature on Socioeconomic Status and Health 2012 Hyattsville, MD 22812021

[B2] Ogden CL Carroll MD Kit BK Flegal KM Prevalence of childhood and adult obesity in the United States, 2011–2012 JAMA 2014 311 8 806 814 10.1001/jama.2014.732 24570244 PMC4770258

[B3] Han JC Lawlor DA Kimm SY Childhood obesity Lancet 2010 375 9727 1737 1748 10.1016/S0140-6736(10)60171-7 20451244 PMC3073855

[B4] Levi J Segal L Thomas K Laurent RS Lang A Rayburn J Fox C, Daruwala N F as in Fat Robert Wood Johnson Foundation, Trust for America’s health 2013

[B5] Singh GK Kogan MD Van Dyck PC Changes in state-specific childhood obesity and overweight prevalence in the United States from 2003 to 2007 Arch Pediatr Adolesc Med 2010 164 7 598 607 20603458 10.1001/archpediatrics.2010.84

[B6] Telama R Yang X Viikari J Valimaki I Wanne O Raitakari O Physical activity from childhood to adulthood: a 21-year tracking study Am J Prev Med 2005 28 3 267 273 10.1016/j.amepre.2004.12.003 15766614

[B7] Haynos AF O’Donohue WT Universal childhood and adolescent obesity prevention programs: review and critical analysis Clin Psychol Rev 2012 32 5 383 399 10.1016/j.cpr.2011.09.006 22681912

[B8] Shrestha P Ghimire L A review about the effect of life style modification on diabetes and quality of life Glob J Health Sci 2012 4 6 185 190 23121755 10.5539/gjhs.v4n6p185 PMC4776966

[B9] Tomporowski PD Davis CL Miller PH Naglieri JA Exercise and Children’s Intelligence, Cognition, and Academic Achievement Educ Psychol Rev 2008 20 2 111 131 10.1007/s10648-007-9057-0 19777141 PMC2748863

[B10] Trudeau F Shephard RJ Physical education, school physical activity, school sports and academic performance Int J Behav Nutr Phys Act 2008 5 10 10.1186/1479-5868-5-10 18298849 PMC2329661

[B11] Lavelle HV Mackay DF Pell JP Systematic review and meta-analysis of school-based interventions to reduce body mass index J Public Health (Oxf) 2012 34 3 360 369 10.1093/pubmed/fdr116 22267291

[B12] Barlow SE Expert committee recommendations regarding the prevention, assessment, and treatment of child and adolescent overweight and obesity: summary report Pediatrics 2007 120 Suppl 4 S164 S192 18055651 10.1542/peds.2007-2329C

[B13] Dobbins M Husson H DeCorby K LaRocca RL School-based physical activity programs for promoting physical activity and fitness in children and adolescents aged 6 to 18 Cochrane Database Syst Rev 2013 2 CD007651 10.1002/14651858.CD007651.pub2 PMC7197501 23450577

[B14] Bandura A Social learning theory 1977 Englewood Cliffs, N.J: Prentice Hall

[B15] McLeroy KR Bibeau D Steckler A Glanz K An ecological perspective on health promotion programs Health Educ Q 1988 15 4 351 377 10.1177/109019818801500401 3068205

[B16] Fishbein M Ajzen I Belief, attitude, intention, and behavior: an introduction to theory and research 1975 Reading, MA: Addison-Wesley

[B17] Leger LA Mercier D Gadoury C Lambert J The multistage 20 metre shuttle run test for aerobic fitness J Sports Sci 1988 6 2 93 101 10.1080/02640418808729800 3184250

[B18] Centers for Disease Control and Prevention Guidelines for school health programs to promote lifelong healthy eating MMWR 1996 45 RR-9 1 41 8637498

[B19] Centers for Disease Control and Prevention Guidelines for school and community programs to promote lifelong physical activity among young people MMWR 1997 46 RR-6 1 36 9072670

[B20] U.S. Department of Agriculture and U.S. Department of Health and Human Services: Dietary Guidelines for Americans, 2010 2010 7 Washington, DC: U.S. Government Printing Office [http://www.health.gov/dietaryguidelines/dga2010/DietaryGuidelines2010.pdf] 10.3945/an.111.000430 PMC3090168 22332062

[B21] Centers for Disease Control and Prevention How much physical activity do children need? 2011 [http://www.cdc.gov/physicalactivity/everyone/guidelines/children.html]

[B22] American Academy of Pediatrics Media and Children[http://www.aap.org/en-us/advocacy-and-policy/aap-health-initiatives/pages/media-and-children.aspx]

[B23] Centers for Disease Control and Prevention Health Education Curriculum Analysis Tool 2012 Atlanta, GA: CDC

[B24] U.S. Department of Health and Human Services Physical Activity Guidelines for Americans 2008 Washington, D.C

[B25] Centers for Disease Control and Prevention A SAS Program for the CDC Growth Charts[http://www.cdc.gov/nccdphp/dnpao/growthcharts/resources/sas.htm]

[B26] Beam M Ehrlich G Donze Black J Block A Leviton LC Evaluation of the healthy schools program: Part I. Interim progress Prev Chronic Dis 2012 9 E65 22380938 10.5888/pcd9.110106 PMC3366696

[B27] Elinder LS Heinemans N Hagberg J Quetel AK Hagstromer M A participatory and capacity-building approach to healthy eating and physical activity- SCIP-school: a 2-year controlled trial Int J Behav Nutr Phys Act 2012 9 145 10.1186/1479-5868-9-145 23245473 PMC3545832

[B28] Fung C Kuhle S Lu C Purcell M Schwartz M Storey K Veugelers PJ From “best practice” to “next practice”: the effectiveness of school-based health promotion in improving healthy eating and physical activity and preventing childhood obesity Int J Behav Nutr Phys Act 2012 9 27 10.1186/1479-5868-9-27 22413778 PMC3414762

[B29] Tomlin D Naylor PJ McKay H Zorzi A Mitchell M Panagiotopoulos C The impact of Action Schools! BC on the health of Aboriginal children and youth living in rural and remote communities in British Columbia Int J Circumpolar Health 2012 71 17999 22456048 10.3402/ijch.v71i0.17999 PMC3417517

[B30] Kropski JA Keckley PH Jensen GL School-based obesity prevention programs: an evidence-based review Obesity (Silver Spring) 2008 16 5 1009 1018 10.1038/oby.2008.29 18356849

[B31] Vouri I Sapkota S Guell C Promoting Physical Activity in Schools: An Important Element of a Health-Promoting School WHO Information Series on School Health 2007 Geneva, Switzerland: World Health Organization

[B32] Jones JT Furner M Health-Promoting Schools: A healthy setting for living, learning and working WHO’s Global School Health Initiative 1998 Geneva, Switzerland: World Health Organization

[B33] Kuczmarski RJ Ogden CL Guo SS Grummer-Strawn LM Flegal KM Mei Z Wei R Curtin LR Roche AF Johnson CL CDC growth charts for the United States: Methods and development. National Center for Health Statistics Vital Health Stat 2002 11 246 12043359

[B34] Welk GJ De Saint-Maurice Maduro PF Laurson KR Brown DD Field evaluation of the new FITNESSGRAM^®^ criterion-referenced standards Am J Prev Med 2011 41 4 Suppl 2 S131 S142 21961613 10.1016/j.amepre.2011.07.011

[B35] Glasgow RE Vogt TM Boles SM Evaluating the public health impact of health promotion interventions: the RE-AIM framework Am J Public Health 1999 89 9 1322 1327 10.2105/AJPH.89.9.1322 10474547 PMC1508772

[B36] HealthMPowers HealthMPowers Overview: PEP Project Overview, Program Outcomes [http://healthmpowers.org/getmedia/0e3a73ff-60a7-4c80-b5ec-74d16d8705ee/Health-M-Powers-Overview-PEP-results.aspx]

